# Drug Transport Mechanism of Oral Antidiabetic Nanomedicines

**DOI:** 10.5812/ijem.8984

**Published:** 2014-01-01

**Authors:** Evren Gundogdu, Aysu Yurdasiper

**Affiliations:** 1 Department of Biopharmaceutics and Pharmacokinetics, Faculty of Pharmacy, Ege University, Izmir, Turkey; 2 Department of Pharmaceutical Technology, Faculty of Pharmacy, Ege University, Izmir, Turkey

**Keywords:** Nanomedicines, Diabetes, Drug Transport

## Abstract

**Context:**

Over the last few decades, extensive efforts have been made worldwide to develop nanomedicine delivery systems, especially via oral route for antidiabetic drugs. Absorption of insulin is hindered by epithelial cells of gastrointestinal tract, acidic gastric pH and digestive enzymes.

**Evidence Acquisition:**

Recent reports have identified and explained the beneficial role of several structural molecules like mucoadhesive polymers (polyacrylic acid, sodium alginate, chitosan) and other copolymers for the efficient transport and release of insulin to its receptors.

**Results:**

Insulin nanomedicines based on alginate-dextran sulfate core with a chitosan-polyethylene glycol-albumin shell reduced glycaemia in a dose dependent manner. Orally available exendin-4 formulations exerted their effects in a time dependent manner. Insulin nanoparticles formed by using alginate and dextran sulfate nucleating around calcium and binding to poloxamer, stabilized by chitosan, and subsequently coated with albumin showed a threefold increase of the hypoglycemic effect in comparison to free insulin in animal models. Solid lipid nanoparticles showed an enhancement of the bioavailability of repaglinide (RG) within optimized solid lipid nanoparticle formulations when compared with RG alone.

**Conclusions:**

Nanoparticles represent multiparticulate delivery systems designed to obtain prolonged or controlled drug delivery and to improve bioavailability as well as stability. Nanoparticles can also offer advantages like limiting fluctuations within therapeutic range, reducing side effects, protecting drugs from degradation, decreasing dosing frequency, and improving patient compliance and convenience

## 1. Context

Type I diabetes is a fast growing epidemic which in 2011 was estimated to affect over 350 million people worldwide, and its prevalence is expected to increase to approximately 550 million by 2030 (1, 2). Type I diabetes occurs when the pancreas fails to produce enough insulin and the insufficient production of insulin causes hyperglycemia. Insulin therapy is commonly delayed despite the harmful consequences, partly due to the inconvenience and complications associated with insulin administration by injection (3, 4). Thus, the development of alternative drug delivery methods for insulin has represented an important concern for clinicians and pharmaceutical companies during the last decade. Nanomedicine represents a branch of medicine focused on developing nanosized molecular vectors for the transport of drug molecules through the patient’s organism to the targeted organ, while entirely conserving the medicine’s therapeutic properties during the transport phase. At present, nanomedicine is a promising drug packing field, which holds a promising future for the improvement of medical diagnoses and therapies. 

Nanomedicines have numerous advantages for the oral drug delivery route. Several disease related drugs are successfully encapsulated in nanomedicines in order to improve bioavailability, bioactivity and control delivery. Especially, these particles are of small size (within the micro or nano range) and capable of encapsulating peptide drugs such as insulin (5). Also, nanoparticles have a high intracellular uptake due to their small size and easy mobility. These novel dosage forms protect them from enzymatic degradation in the adverse gastrointestinal (GI) environment, while also enabling easy transport and improving the pharmacokinetics, bioavailability and therapeutic efficacy after administration (6-8). Remarkable and extensive reviews on oral insulin delivery systems adopting various approaches exist in the literature, including numerous on nanomedicines. In the present review, the recent developments and current approaches in drug transport and metabolism characteristics of oral antidiabetic nanomedicine will be considered.

### 1.1. Why Oral Antidiabetic Drug Delivery?

Currently, multiple daily subcutaneous injections of insulin are the standard treatment for insulin-dependent diabetic patients. Nevertheless, clinical studies showed that a significant percentage of patients failed to attain lasting glycemic control on insulin treatment (9, 10). Well-recognized reasons for this failure are the poor compliance in patients who are afraid of injection and the physiological reasons related to parenteral administration. The objective of pharmaceutical formulations is the transformation of drug compounds into active products with the desired therapeutic effect. During the last decade, investigators have shown a strong interest for developing a delivery system for the oral administration of insulin. Present research initiatives in this domain are beginning to get closer to viable solutions for oral insulin treatment in diabetic patients. Although oral administration has the best compliance and it takes advantage of a portal-hepatic delivery (11), there are several limitations to delivering insulin by oral route. The inactivation of the hormone by enzymatic digestion in the stomach and intestine and the poor permeability of the intestinal epithelium for insulin, owing to its high molecular weight and lack of lipophilicity, are responsible for a the low oral bioavailability of insulin.

Lassman-Vague and Raccah (12) reviewed the obstacles of antidiabetic drug administration especially for insulin in different delivery routes. Delivery of insulin via the ocular route was tested in animal models in combination with different absorption enhancers. Vaginal and rectal routes have been investigated, but the absorption rate and bioavailability are poor due to the thick mucosal layers in these tissues. Nasal delivery has also been evaluated because of easy access and large absorption area associated with this route. Unfortunately, the highly active mucociliary clearance in the nose hindered drug absorption resulting in poor bioavailability. Compared to this administration, oral and sublingual insulin administration provides better results (13). Taking all these facts into account, the oral route is considered to be the most feasible and convenient method of drug administration to improve compliance among diabetic patients. When the insulin is administered by oral route, it is absorbed directly from the intestine and then transported to the liver via the portal circulation, where it inhibits hepatic glucose production (14). Unlike other delivery routes, the gut is the natural pathway of nutrient absorption surface of all routes and should theoretically provide a better sustainability (15, 16).

## 2. Evidence Acquisition

## 2.1. Nanomedicines and nanoparticles

Oral formulations have some potential advantages and face several common problems, particularly for peptides and proteins: poor stability in the gastric fluid, low solubility/bioavailability and the mucus barrier can prevent drug penetration and absorption. Nanoparticle formulations are being developed to encapsulate and protect drugs and release them in a controlled manner to overcome these limitations (17, 18). Nanoparticles have varying shapes, ranging in size from 10 to 1000 nm. Their small size allows for a higher surface area to volume ratio and therefore provides a higher adsorption capacity for surface loading (19). The advantages of using nanoparticles include protection of drug, peptide, or other contents from degradative enzymes, increased mucoadhesion and increased retention in the gastrointestinal tract. Increased mucoadhesion through the use of nanoparticles has the benefit of improving the oral delivery of poorly adsorbed drugs, proteins, and other contents by increasing the time and amount of interaction with the mucus layer of the intestine. It is hypothesized that this increased mucosal interaction is explainable through electrostatic interactions between the positively charged nanoparticles and the negatively charged mucus and endothelial layer, or through a physical capture of the nanoparticle by the mucus layer. Nanoparticles can have increased mucoadhesive properties with the use of mucoadhesive polymers, which include derivatives such as Eudragit (Evonik, Essen, Germany), poly (acrylic acid), sodium alginate, and chitosan. Although the mucoadhesive properties can be beneficial, they can also provide a means of quick exit if the nanoparticles become associated with the loosely attached mucus layer which is rapidly shed by the stomach. Therefore it is preferred to achieve attachment in the deeper mucus layer, which is shed less often and provides a longer interaction between the nanoparticle and the gastrointestinal tract (20, 21).

The absorption mechanisms of orally delivered drug loaded nanoparticles have attracted less attention than their design. The design of new nanoparticles for oral administration usually focuses on overcoming the different barriers in the gastrointestinal tract. The nanoparticles must resist the harsh gastrointestinal environment, e.g the low pH in the stomach and the degradative enzymes. However, the major barrier to their absorption remains the intestinal mucosa (22). In order to improve the stability of drugs in the gastrointestinal tract, effective, simple and safe nanoparticle systems have been designed which address the problem of poor drug permeability by numerous ways. Hence, industry may use nanoparticle systems for oral drug delivery in the future, after improving pharmacological effects in comparison with the standard formulations of today.

### 2.2. Nanomedicine Based Systems for Diabetes Patients

The concept of nanomedicine named in 2004 by the European Science Foundation, represents a new area in the field of drug delivery research concerning drug delivery vehicles (6). Recently nanomedicine based oral drug delivery systems have gained an important attention and it is well understood that the nanosize played an important role in the improvement of pharmacological availability. In addition, nanomedicine based systems provide excellent protection to insulin and maintain its stability in physiological fluid, resulting in controlled drug release.

## 3. Results 

A number of reviews exist on nanomedicine based oral insulin delivery systems (23, 24). The permeation enhancers and enzyme suppressors for oral drug delivery of insulin have been investigated in previous studies (25, 26), but a major approach is to carry insulin within nanoparticles. One of them is that nanoparticulate systems encompass solid biodegradable nanoparticles (27). A limited number of polymers can be used as constituents of nanoparticles designed to deliver drugs in vivo. Commonly used polymers for insulin delivery are chitosan/alginate (28), poly (lactide-co-glycolic acid) (PLGA) (29) and copolymers including one part of poly (ethylene glycol) (30). Shelesh et al. (31) has aimed to develop glipizide (GPZ) loaded biodegradable nanoparticles by using a biodegradable polymer, PLGA, as a sustained release carrier. In general, rapid gastrointestinal absorption is required for oral hypoglycemic drugs, to prevent a sudden increase in the blood glucose level after food intake in patients with diabetes mellitus. The gastrointestinal absorption rate of GPZ appears to be rather slow in conventional dosage form (i.e. tablets) (32).

Reis et al. (6) developed and evaluated the efficacy of a novel oral insulin nanomedicine system based on alginate-dextran sulfate core with a chitosan-polyethylene glycol-albumin shell. They observed that when the insulin loaded nanoparticles were administered orally to diabetic rats, they reduced glycaemia in a dose dependent manner. Exendin-4 is a potent insulinotropic agent in diabetic patients. However, its therapeutic utility is limited due to the frequent injections required. In a study, Nguyen et al. (33) developed an oral exendin-4 by using an enteric-coated capsule containing pH-responsive nanoparticles. These nanoparticles were labeled with iodine (I). After oral administration of I-labeled-exendin-4 loaded nanoparticles in rats, the biodistribution of the administered drug was investigated using a SPECT/CT scanner. They found that the radioactivity of I-exendin-4 propagated from esophagus, stomach and small intestine and then absorbed into the systemic circulation, in a time dependent manner. The results suggest that orally available exendin-4 formulations show great promise as a potential therapy for diabetic patients.

In one study, insulin nanoparticles were formed by using alginate and dextran sulfate nucleating around calcium and binding to poloxamer, stabilized by chitosan, and subsequently coated with albumin and evaluated in streptozotocin-induced Wistar diabetic rats. Pharmacodynamic and pharmacokinetic parameters were evaluated at a dose of 50 IU/kg nanoencapsulated insulin, and the 13% oral bioavailability represented a threefold increase in comparison to free insulin. Therefore, the nanoparticles facilitated the oral delivery of insulin, and potentially that of other therapeutic proteins (34). Rawat et al. (35) formulated the new solid lipid nanoparticles of repaglinide (RG) for oral drug delivery and evaluated them in terms of bioavailability of RG. They found that the relative bioavailability of RG was enhanced with optimized solid lipid nanoparticle formulations when compared with RG alone. Furthermore, the in vitro toxicity study indicated that the solid lipid nanoparticles were well tolerated.

### 3.1. Drug Transport Mechanism of Oral Antidiabetic Drugs

Hydrophilic drugs and proteins are slowly and incompletely passively absorbed and distribute poorly into the cell membrane. The transport of proteins across the intestinal wall may take place via various pathways (36). The transport can occur primarily through the cell membrane of the enterocytes (transcellular transport) or via the tight junctions between the cells (paracellular transport). Therefore, it is assumed that these drugs are transported through the water filled pores of the paracellular pathway across the intestinal epithelium. However, it is not established whether or not these drugs are transported partly by the transcellular route. The transcellular passive diffusion pathway is mostly limited to drugs that are non-polar, are lipid soluble, and are not electrically charged at the physiological pH of the small intestinal lumen. When it is considered hydrophilic, a drug molecule has a partition coefficient between the cell membrane and the extracellular fluid (Pmembr) of 1 × 10^-3^, i.e. a log Pmembr of -3. For comparison, the log octanol/water partition coefficient (log Poct) for molecules assumed to be transported by the paracellular route (e.g. mannitol) is also in the order of -3. Then it is assumed that the surface area of the luminal cell membrane of the intestinal epithelium is 1000-fold larger than that of the paracellular space. The larger surface area of the cell membrane will compensate for the difference in partitioning between the cell membrane and the extracellular fluid. As a result, it could be thought that the hydrophilic drug is transported in equal amounts by the paracellular and transcellular routes. However, in reality, the tight junctions which gate the entrance to the paracellular pathway restrict the paracellular transport of drugs even further (37-39).

The low efficiency of the paracellular pathway has stimulated investigations into ways to enhance the permeability by this route. Many of these studies have been performed in monolayers of intestinal epithelial cells and have provided new insight into the regulation of tight junctions (the rate limiting barrier) of the paracellular pathway (37, 39). Insulin has low lipophilicity with a log Poct of about 0.0215. Furthermore, the iso-electric point of insulin is around five and because of that, insulin is negatively charged at the neutral pH of the small intestine. Thus, the entry into the cell membrane is unfavorable. The primary pathway is available for transport of insulin across the epithelium by aqueous paracellular pathway (40, 41).

Insulin receptors have been identified in the basolateral membranes of dog intestinal mucosa, in the mouse intestinal cells and in the membrane of Caco-2 cells (42). Kendzierski et al. (43) analyzed the ability of the gut to make insulin. It was suggested that the insulin receptors might play an autocrine or paracrine role for the insulin synthetized in the gut. Intracellular immunoreactivity towards insulin was found in glandular cells of the stomach and colon, but no immunoreactivity was observed in the small intestine. Several studies with healthy volunteers or patients revealed that the time to reach peak serum GPZ concentration ranged from 0.5 to 1 hour following oral administration of the GPZ tablet. Slow absorption of the drug usually originates from the poor permeability of the drug across the GI membrane. The dose of GPZ is 5 mg tid, and hence there is always a need for the development of a sustained release formulation of GPZ. 

## 4. Conclusions

This review mainly focused on the nanomedicines and their transport mechanism of antidiabetic drugs. Nanoparticles are multiparticulate delivery systems designed to obtain prolonged or controlled drug delivery and to improve bioavailability as well as stability. Nanoparticles can also offer advantages like limiting fluctuations within therapeutic range, reducing side effects, protecting drugs from degradation, decreasing dosing frequency, and improving patient compliance and convenience (31). From the very recent reports on oral delivery systems, it is obvious that an important focus is on polymeric nanomedicine drug delivery systems. Multifunctional nanomedicines, which can enhance the insulin absorption by transcellular or paracellular pathway and prolong the gastrointestinal retention, hold the basis for improving bioavailability. By taking advantage of nanomedicines for oral delivery, it is hoped to reach the goal of the much awaited successful oral insulin formulation.
